# On the Origin and Progress of Renal Surgery

**Published:** 1899-09

**Authors:** 


**On the Origin and Progress of Renal Surgery.** By HENRY MORRIS, M.B. Pp. viii., 288. London Cassell and Company, Limited. 1898.

The Hunterian Lectures which Mr. Morris delivered in 1898 form the greater portion of this admirable volume, which is about equally divided between descriptive matter and tabulations of cases. It is impossible to praise too highly this masterly exposition of the recent advances in renal surgery, which is embellished by excellent figures. It is indicative of an immense amount of labour and research, as well as of personal experience. Although every section is of importance to the surgeon, that dealing with the surgery of the ureter is particularly so, since it emphasises the most important of recent advances. The Heinecke-Mikulicz suture for the relief of ureteral stricture, and the methods of ureteral anastomosis, are deserving of careful study.

Mr. Morris's theory, like his practice, is of a distinctly conservative type; and as he was one of the pioneers in boldly attacking the kidney, so he is the foremost in advocating conservative methods. Surgeons and patients will alike benefit from a study of the work.

